# The Pandemic Experience in Southeast Asia: Interface Between SARS-CoV-2, Malaria, and Dengue

**DOI:** 10.3389/fitd.2021.788590

**Published:** 2021-11-18

**Authors:** Christina Yek, Vu Sinh Nam, Rithea Leang, Daniel M. Parker, Seng Heng, Kimsan Souv, Siv Sovannaroth, Mayfong Mayxay, Sazaly AbuBakar, R. Tedjo Sasmono, Nhu Duong Tran, Hang Khanh Le Nguyen, Chanthap Lon, Kobporn Boonnak, Rekol Huy, Ly Sovann, Jessica E. Manning

**Affiliations:** 1 Department of Critical Care Medicine, National Institutes of Health Clinical Center, Bethesda, MD, United States,; 2 Laboratory of Malaria and Vector Research, National Institute of Allergy and Infectious Diseases, National Institutes of Health, Rockville, MD, United States,; 3 National Institute of Hygiene and Epidemiology, Hanoi, Vietnam,; 4 Ministry of Health, Phnom Penh, Cambodia,; 5 Department of Population Health and Disease Prevention, University of California, Irvine, Irvine, CA, United States,; 6 Department of Epidemiology, University of California, Irvine, Irvine, CA, United States,; 7 Lao-Oxford-Mahosot Hospital-Wellcome Trust Research Unit, Microbiology Laboratory, Mahosot Hospital, Vientiane, Laos,; 8 Institute of Research and Education Development, University of Health Sciences, Vientiane, Laos,; 9 Centre for Tropical Medicine and Global Health, Nuffield Department of Medicine, University of Oxford, Oxford, United Kingdom,; 10 WHO Collaborating Center for Arbovirus Reference and Research (Dengue) and Tropical Infectious Diseases Research and Education Center, Universiti Malaya, Kuala Lumpur, Malaysia,; 11 Eijkman Institute for Molecular Biology, Jakarta, Indonesia,; 12 International Center of Excellence in Research, National Institute of Allergy and Infectious Diseases, National Institutes of Health, Phnom Penh, Cambodia,; 13 Department of Microbiology and Immunology, Faculty of Tropical Medicine, Mahidol University, Bangkok, Thailand

**Keywords:** SARS-CoV-2, COVID-19, malaria, dengue, Southeast Asia

## Abstract

Southeast Asia (SEA) emerged relatively unscathed from the first year of the global SARS-CoV-2 pandemic, but as of July 2021 the region is experiencing a surge in case numbers primarily driven by Alpha (B.1.1.7) and subsequently the more transmissible Delta (B.1.617.2) variants. While initial disease burden was mitigated by swift government responses, favorable cultural and societal factors, the more recent rise in cases suggests an under-appreciation of prior prevalence and over-appreciation of possible cross-protective immunity from exposure to endemic viruses, and highlights the effects of vaccine rollout at varying tempos and of variable efficacy. This burgeoning crisis is further complicated by co-existence of malaria and dengue in the region, with implications of serological cross-reactivity on interpretation of SARS-CoV-2 assays and competing resource demands impacting efforts to contain both endemic and pandemic disease.

## INTRODUCTION

**FIGURE 1 |:**
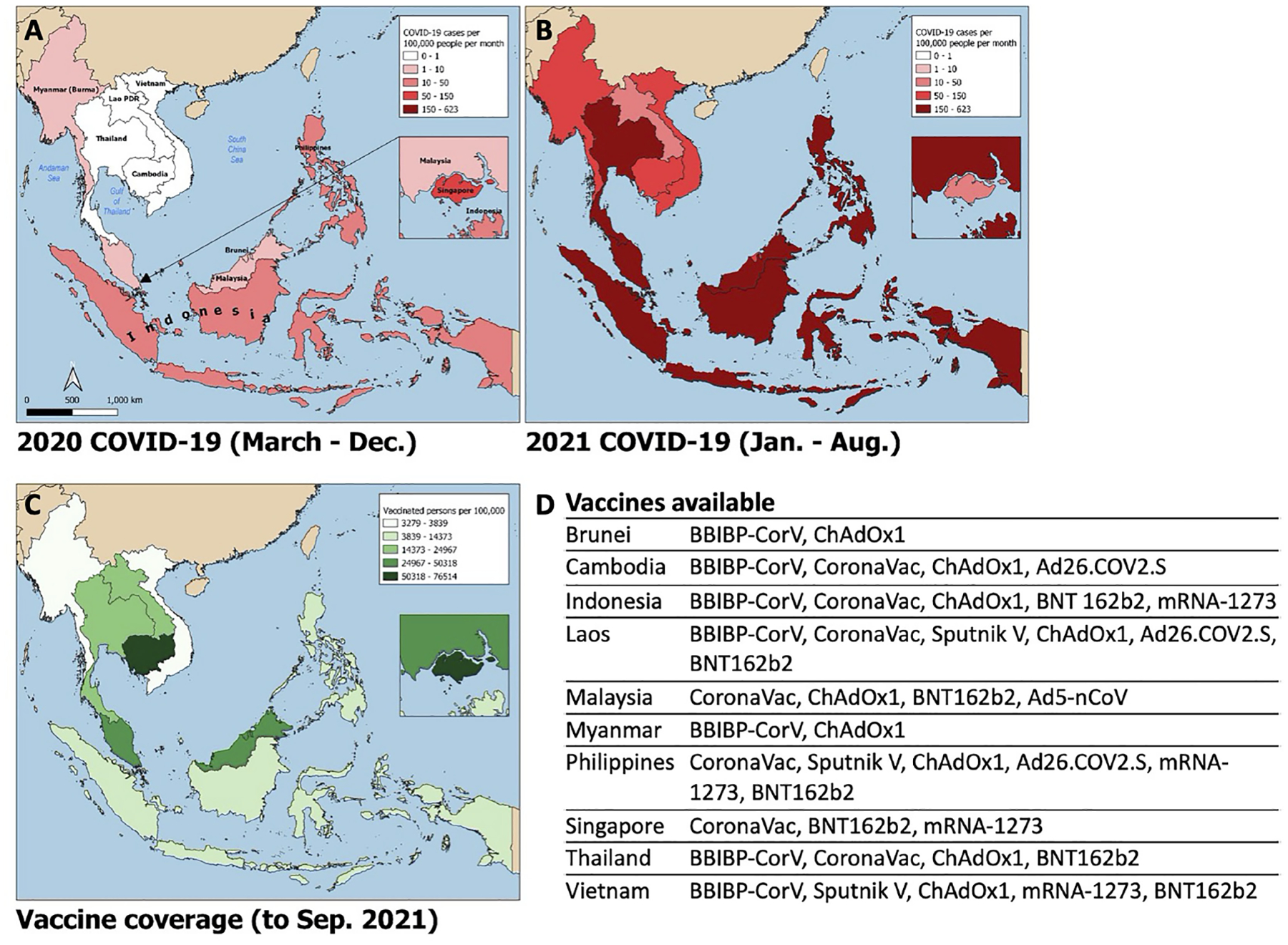
Choropleth maps of Southeast Asia displaying i) average monthly incidence of SARS-CoV-2 infections (new cases per 100,000 people) from March – December 2020 **(A)** and January – August 2021 **(B)**; ii) SARS-CoV-2 vaccine coverage (fully vaccinated persons per 100,000 people; panel **C**) and list of publicly available vaccines by country **(D)**. Data obtained from the Global Change Data Lab (URL: https://covid.ourworldindata.org/, accessed September 9, 2021).

Since the emergence of SARS-CoV-2 in Wuhan, China in December 2019, there have been over 200 million cases of COVID-19 worldwide, with over 4 million confirmed deaths by mid-2021 (1). Despite its proximity to China and global connectivity, Southeast Asia (SEA) experienced relatively low case numbers during the first year of the pandemic (Figure 1A). However, as of July 2021, cases in SEA are surging and the region is now on track to become the next global pandemic hotspot.

SEA is a geopolitical region spanning approximately 1.7 million square miles from its northern borders with India and China through the tip of the Indonesian archipelago in the south. In this paper we define SEA as the ten member countries of the Association of Southeast Asian Nations (ASEAN), consisting of Brunei, Cambodia, Indonesia, Laos, Malaysia, Myanmar, the Philippines, Singapore, Thailand, and Vietnam. The region is home to over 650 million people, with 3.5% of the world’s population in Indonesia alone (2). Climate is tropical and landscape is diverse, ranging from the densely populated metropoles of Manila and Jakarta to rural communities bordering the world’s oldest rainforest in Borneo. Its location along historic trade routes and young population feeding into an inexpensive, plentiful labor force have led to SEA’s development as a global hub of commerce and industry. Economic and population growth have in turn spurred rapid high-density urbanization, changes in land use, internal and cross-border migration, and socioeconomic inequities that prime for the emergence of novel and resurgent infectious diseases (3).

In this review, we provide a perspective of how the SEA experience of SARS-CoV-2 has been molded by the region’s unique infectious disease exposures. We predict that the latest wave of the pandemic will have both significant direct implications on population health in the region and vast indirect effects *via* the subversion of fragile systems that contain, manage, and monitor endemic diseases such as dengue and malaria.

## THE COVID-19 PANDEMIC: SOUTHEAST ASIAN EXPERIENCE

**FIGURE 2 |:**
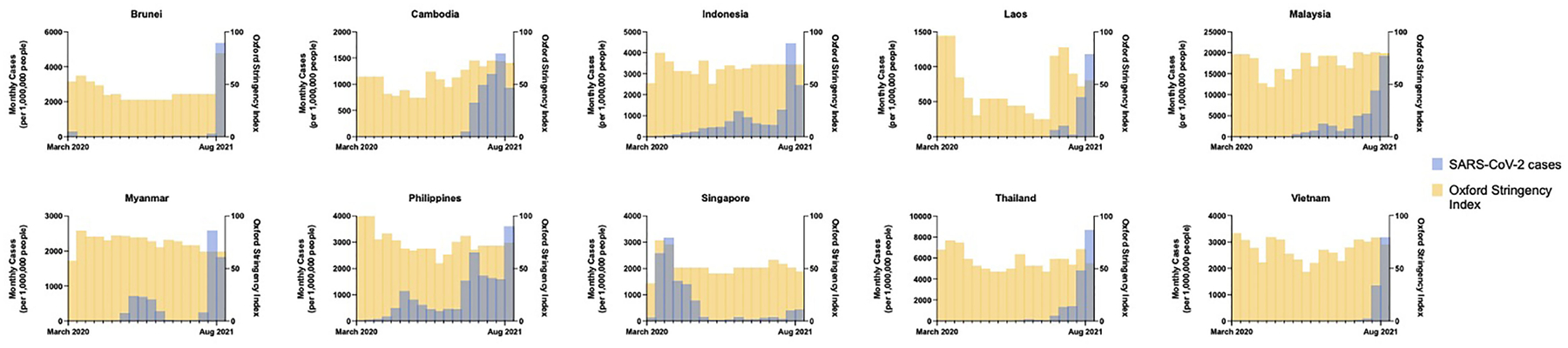
Monthly incidence of SARS-CoV-2 infections (new cases per 1,000,000 people) showing the effects of government COVID-19 pandemic control measures (Oxford Stringency Index) from March 2020 through August 2021 by country. Data obtained from the Global Change Data Lab (URL: https://covid.ourworldindata.org/, accessed September 9, 2021).

SARS-CoV-2 spread quickly from neighboring China in 2020, with index cases reported in Thailand, the Philippines, and Singapore by mid-January (4–6). The majority of countries in SEA quickly implemented strict border control and lockdown policies in early 2020 including movement restriction, mandatory quarantine for international arrivals, isolation of confirmed positive cases or contacts thereof, shutdown of schools and non-essential businesses, mandated mask-wearing, widespread testing, aggressive contact tracing, and public education campaigns (4–9). Pandemic control measures may have been further augmented by favorable pre-existing cultural and societal factors including open-air societies with a young demographic, lack of mass transit, public compliance with government mandates, and intact public health surveillance and response systems from prior epidemics such as H5N1 and SARS-CoV-1. There was inevitably intraregional variability in pandemic control (Figure 2). Despite stringent lockdown policies, Singapore experienced a wave of cases in April through June 2020 driven by a surge in transmission among foreign workers housed in closed quarters (10). The Philippines experienced an initial wave that peaked in August 2020, while Indonesia and Malaysia saw smaller waves at year-end, largely attributable to easing of movement restrictions. The true scale of the problem may have been underestimated in several countries due to case under-reporting related to poor testing infrastructure (11) and civil unrest (12). Even so, a few serosurveys conducted during the second half of 2020 demonstrated relative population naivety (13, 14). While the Western world was swept by devastating case numbers and fatalities, SEA seemed to emerge from the 2020 pandemic year relatively unscathed.

In April 2021, India experienced a surge in SARS-CoV-2 cases, which at the time represented over half of daily cases worldwide. A novel variant, Delta (B.1.617.2), initially identified in Maharashtra, India in December 2020, was linked to the large wave of cases there (15) with subsequent spread to the rest of the globe (16). Delta is highly transmissible and more pathogenic than prior variants of concern. Additionally, there is seemingly reduced efficacy of currently available vaccines to the Delta variant (17). Paired with waning immunity from vaccination and natural infection over time (18), this ‘perfect storm’ contributed to the latest pandemic wave from which SEA has not been spared (Figure 1B). SARS-CoV-2 cases began rising in Indonesia and Malaysia in late May 2021, shortly after the Muslim Eid-Al-Fitr holiday. The rest of the region quickly followed; SEA is now experiencing an average number of daily cases over twenty times that seen in the same period of the prior year and approximately eight times that seen at the highest point of the pandemic to date. The Delta variant constitutes the bulk of cases in Vietnam, Myanmar and Singapore, and rising case numbers in Malaysia, Thailand, Cambodia, and Indonesia. Alpha (B.1.1.7) and Beta (B.1.351) variants are of prominence as well in Thailand and Cambodia, and Indonesia, Malaysia, and the Philippines, respectively. Phylogenetic studies further suggest these variants had likely been circulating unchecked in the community and across borders for months (19).

Possible contributors to the recent surge could include premature easing of restrictions due to complacency following earlier success of pandemic control measures, and under-recognition of the potential impact of novel SARS-CoV-2 variants. Financial considerations may have factored strongly in SEA’s lower-income members, where existing economic margins were insufficient to bolster the impact of prolonged lockdown. This has been further compounded by the slower-than-desired vaccine rollout in the region; country-specific population coverage in SEA currently averages 17%, ranging from 5% in Myanmar to 77% in Singapore (Figure 1C). A variety of vaccines are in use including several for which Phase III clinical trial data are lacking (20, 21), WHO approval has not been issued for emergency use (22), or of which there is off-label pediatric use (23) (Figure 1D). Additional fault has been placed on the movement of undocumented migrant workers across porous borders particularly in the Greater Mekong Subregion consisting of Myanmar, Thailand, Cambodia, Laos, and Vietnam (24, 25). Myanmar, which has experienced an upsurge of political unrest and civil conflict including a military coup in February 2021, is undergoing a humanitarian crisis that undermines the country’s ability to deal with its current health crisis (12). To tackle the latest pandemic wave, several countries implemented or maintained lockdown measures comparable to those in 2020, as measured by the Oxford Stringency Index (Figure 2). By August 2021, Cambodia had successfully vaccinated 90% of the adult population and had already begun an aggressive campaign to administer booster shots (26). It remains to be seen whether these measures will be sufficient to curb the crisis in the region.

## SOUTHEAST ASIA – AT THE CONFLUENCE OF ENDEMIC, EMERGING, AND RESURGENT INFECTIOUS DISEASES

SEA has a substantial burden of infectious disease, driven mainly by respiratory and intestinal diseases but with significant representation from tuberculosis, HIV, malaria, and arboviruses (27). Large populations of biodiverse wildlife serve as reservoirs for emerging infections, while close geographic and cultural interfaces between man and nature, such as through deforestation for commercial use and subsistence farming, wildlife trade and consumption, and application of bat guano in local agricultural practices, predispose to transmission of vector-borne and zoonotic disease (28). Malaria is endemic to most countries in SEA, with local autochthonous transmission in Indonesia, Myanmar, Thailand, Cambodia, Laos, Vietnam, and the Philippines. Average case incidence in these countries approximated 11 cases per 1,000 population at risk in 2010. Numbers have since decreased by over 80% in the past decade, attributable to collaborative regional eradication efforts such as the Mekong Malaria Elimination Program (29). Even as cases of *Plasmodium falciparum* diminish, there has been a rise in zoonotic malaria. Malaysia, which saw no cases of human malaria in 2018 and 2019, reported over 3000 annual cases of *P. knowlesi* in both years, from 1600 just two years prior (29). *P. cynomolgi* has also made the jump into human hosts with cases of natural infection described in Thailand, Cambodia, and Malaysia in the last decade. A host of other simian species has been identified with abundance in local macaques, including some with demonstrable *in vitro* infectivity of human cells and shared vectors with known human pathogens (30). Dengue is another prominent vector-borne disease in SEA, where prevalence ranks in the top quintile amongst WHO geographic regions. Climate change and changing land-use patterns may be contributing to rising cases over the past decade; 2019 saw unprecedented dengue outbreaks in Malaysia, Indonesia, the Philippines, Thailand, Vietnam, Cambodia and Laos akin to what was simultaneously reported in the Americas and South Asia (31). Other vector-borne diseases in the region such as chikungunya, Zika, Japanese Encephalitis Virus, and tickborne diseases such as *Rickettsia* spp. and *Orientia* spp. are similarly projected to increase over the coming years (32, 33). Several zoonoses arising in SEA have also come to recent attention due to the severity of their associated clinical syndromes, and/or their pandemic potential. Over the past two decades these have included influenza viruses, orthoreoviruses, Nipah virus, zoonotic malaria, and *Streptococcus suis*, amongst others (27, 34). SEA has also seen its share of pandemics with H5N1, H1N1, SARS-CoV-1, and now SARS-CoV-2 sweeping the region, in part due to strong human movement linkages and shared borders with global powerhouses China and India. Finally, unchecked antibiotic use in both humans and livestock has led to the rise of drug resistant pathogens in SEA. Multi-and extensively drug-resistant tuberculosis has been detected in high-burden countries including the Philippines, Myanmar, and Thailand (27). Artemisin-resistant *P. falciparum* was first detected along the Thai-Cambodia border and continues to circulate in the region (32, 35). More recently, the WHO identified SEA as a neglected hotspot for the emergence of antibiotic resistance in common bacterial pathogens including *Staphylococcus aureus, Streptococcus pneumoniae, Neisseria gonorrhoeae*, and several gram-negative bacteria (36). This unique milieu of endemic, emerging, and resurgent infections in SEA makes it an ideal model for understanding the interface between tropical diseases and the rising threat of SARS-CoV-2.

## PRIOR EXPOSURE TO ENDEMIC DISEASES MAY INFLUENCE SARS-COV-2 SERO-ASSAYS AND PRE-EXISTING IMMUNITY

While the mainstay of SARS-CoV-2 diagnostics involves nucleic acid testing, the role of serologic testing is increasingly recognized with applications including for rapid diagnosis in resource-scarce settings, measurement of vaccine response in vaccine efficacy studies, and population-wide serosurveys, amongst others (37). However, serologic assays can be affected by cross-reactivity leading to false positive results, and calibration to target populations is important to optimize test performance. High rates of background reactivity of SARS-CoV-2 seroassays have been noted in sub-Saharan Africa (38–42) with postulated mechanisms including cross-reactivity with endemic human coronaviruses or polyclonal B-cell activation from unrelated infections such as malaria, cytomegalovirus, and Epstein Barr virus. Pre-pandemic SARS-CoV-2 seroreactivity was also recently demonstrated in malaria-experienced individuals from Cambodia (43), although a second smaller study of pre-pandemic sera from Vietnam found no positive samples (44).

Most studies of seasonal human coronaviruses (hCoVs) have demonstrated some level of cross-reactivity with SARS-CoV-2 seroassays, with (45) or without (46, 47) demonstrable *in vitro* viral neutralization activity of identified antibodies. Cross-reactive humoral immunity among sarbecoviruses has also been found (48) although notably the two human sarbecoviruses do not appear to demonstrate significant cross-neutralization, potentially due to the rarity of cross-neutralizing antibodies (48–51). The ubiquitous hCoVs 229E, OC43, NL63, and HKU1 are endemic to SEA (52), but unique to the region is a high prevalence of coronaviruses circulating in zoonotic reservoirs, including several recently identified sarbecoviruses with high sequence homology to SARS-CoV-2 in *Rhinolophus* spp. bats and *Manis* spp. pangolins in Thailand, Laos and Cambodia (53–56). Population-level serological studies of exposure to these zoonotic coronaviruses are lacking, though transmission has been detected in individuals with high levels of wildlife exposure including forest workers, farmers applying bat guano in agricultural practices, and in suppliers and consumers of wildlife (57, 58). Finally, SEA was also affected by SARS-CoV-1 during the 2002–2003 outbreak; however, case numbers were low, making this an unlikely influencer of subsequent SARS-CoV-2 transmission dynamics (59). Ultimately, assay interpretation may benefit from simultaneous testing for antibodies to multiple coronaviruses to identify co-variation that could better differentiate serologic indicators of prior exposure from false positives related to cross-reactivity (60, 61).

Cross-reactivity of SARS-CoV-2 seroassays has also been described with prior exposure to malaria. In a recent study of 528 malaria-experienced Cambodians, analysis of pre-pandemic samples found 13.8% positivity of non-neutralizing IgG to SARS-CoV-2 spike and RBD antigens, with higher levels of antibodies to a *P. falciparum* antigen in SARS-CoV-2 seropositive as compared to seronegative populations (43). Acute malaria has been found to trigger a dysregulated polyclonal B cell expansion that can lead to cross-reactivity with a number of serologic assays for unrelated conditions (62–64). Additionally, there may be shared epitopes between SARS-CoV-2 and *Plasmodium* spp. that could further explain production of cross-reactive antibodies (65–67), though these appear to be non-functional (43).

Initial case reports also suggested false positivity of SARS-CoV-2 assays in patients with acute dengue (68, 69). Larger studies examining the fidelity of commercially available lateral flow assays for IgG/IgM against SARS-CoV-2 showed low levels of cross-reactivity in dengue-experienced patients ranging from to 2.3 to 7.5% (70–72). One study applied a SARS-CoV-2 IgG chemiluminescence assay to 84 samples from Brazilians with dengue and found no cross-reactivity (73), while a second applied two enzyme immunoassays for SARS-CoV-2 IgG or IgA and found 22% cross-reactivity (as compared to 4% false positivity in non-dengue infected controls) (74). As with malaria, shared epitopes between the two viruses have been proposed, though data are sparse (74). The variability in performance of different serologic testing methods adds a further layer of complexity to the issue (75).

Lack of assay specificity and region-specific optimization could confound assessments of disease burden and vaccine response in regions where these diseases are endemic, with downstream implications on contact tracing, vaccine rollout, and pandemic preparedness planning. Beyond false positivity of serologic assays it is unclear whether pre-existing immunity may also play a role in mitigating disease burden. Some clinical studies of patients with hCoVs preceding SARS-CoV-2 infection suggest that cross-reactive humoral immunity could play a role in ameliorating disease severity (76, 77), though data is lacking and less still is known about the role of cellular immunity. Though intriguing, the theory of pre-existing immunity is perhaps less convincing now in the face of the latest pandemic wave sweeping through SEA.

## COVID-19 AND ITS IMPACT ON TROPICAL DISEASE MANAGEMENT

The latest surge of SARS-CoV-2 in SEA coincides with the southwesterly monsoon season that spans May to September, when warm and wet weather conditions predispose to the propagation of vector-borne diseases including malaria, dengue, and chikungunya. Imposition of lockdown measures presumably increased incidence of vector-borne disease due to the efflux of city workers to residential areas with higher levels of transmission, and cessation or modification of typical vector control measures. This phenomenon may have contributed to the historic dengue outbreak in Singapore in 2020 (78, 79), although incidence patterns in other countries appeared unaffected by SARS-CoV-2 (Supplementary Figure). Regardless, the threat of overlapping epidemics in the coming months is concerning. Outside of Singapore and Brunei, SEA consists of larger low- and middle-income countries in which healthcare infrastructure is heterogeneous and often not built to sustain robust disease surveillance and may not withstand a sudden influx of acutely ill individuals (80). The clinical semblance of acute flavivirus infection with COVID-19 and cross-reactivity of seroassays used for rapid diagnostics pose additional challenges to clinicians and hospital systems that are pressed to develop triage processes to quickly distinguish the two (81, 82).

Other effects of the SARS-CoV-2 pandemic could be more insidious – the diversion of resources to tackle SARS-CoV-2 is expected to detract public and government attention on endemic disease control and surveillance programs, subverting fragile progress that the region has seen over the past decade in terms of malaria control (83, 84). Management of chronic infectious disease such as HIV and tuberculosis, in addition to primary prevention measures including immunization outreach, may also be affected. The unevidenced repurposing of antimalarial and antiretroviral medications for SARS-CoV-2, and overuse of antibiotics for the severely ill, are ongoing threats that could encourage the development of drug resistance in a region already struggling with multidrug resistant *P. falciparum* and bacterial pathogens including carbapenem-resistant *Acinetobacter baumannii, Pseudomonas aeruginosa*, and *Enterobacterales* (32, 35, 36). Finally, the disruption of ongoing vector and ecologic surveillance studies could delay the detection of significant emerging infections, including novel SARS-CoV-2 variants (85). A limitation of this review on the pandemic’s impact on tropical diseases may be that it is too premature, with the true magnitude only assessable many years from now.

## CONCLUSION

SARS-CoV-2 is the latest health crisis to arrive in SEA. The economic, political, and humanitarian toll forecasted are vast. If the region is to survive this threat, it will need to ramp up current efforts. First, vaccine rollout needs to improve in SEA countries with low vaccination rates, although this issue is likely to be ameliorated with increased manufacturing capacity, boosted availability, and delivery of efficacious vaccines in an equitable manner. Next, enhancements to pandemic disease surveillance systems are needed to enable accurate SARS-CoV-2 case detection and reporting, mapping of disease spread, and directed allocation of resources. Existing healthcare infrastructure must be reinforced to prepare for an influx of large numbers of severely ill patients, including with systems in place to care for foreign laborers and immigrants, regardless of documentation or legal status. Finally, shared physical borders and economic interdependence demand that countries in the region forge a common platform, such as through ASEAN, to enable sharing of medical resources, data transference, unified messaging, and establishment of tolerant trade and migration policies to preserve joint growth and ensure the region’s economic and political survival (86).

## Supplementary Material

Supplementary Figure **Supplementary Figure |** Monthly totals of new cases of SARS-CoV-2, malaria, and dengue from March 2020 through August 2021. Data on SARS-CoV-2 obtained from the Global Change Data Lab (URL: https://covid.ourworldindata.org/, accessed September 9, 2021). Data on malaria and dengue obtained from individual country Ministry of Health public surveillance systems; this was only available for Cambodia and Vietnam (both malaria and dengue data available), Laos (malaria data only), and Malaysia and Singapore (dengue data only).

## Data Availability

Publicly available datasets were analyzed in this study. This data can be found here: COVID data obtained from https://covid.ourworldindata.org/data/owid-covid-data.csv. Malaria and dengue national surveillance data obtained from Cambodia, Vietnam, Laos, Malaysia, and Singapore respective Ministries of Health.
