# Supplementary figures and images for: The Pandemic Experience in Southeast Asia: Interface Between SARS-CoV-2, Malaria, and Dengue
Source: Front Trop Dis. Author manuscript; Available in PMC 2022 Apr 1. (PMC8975143; doi:10.3389/fitd.2021.788590)

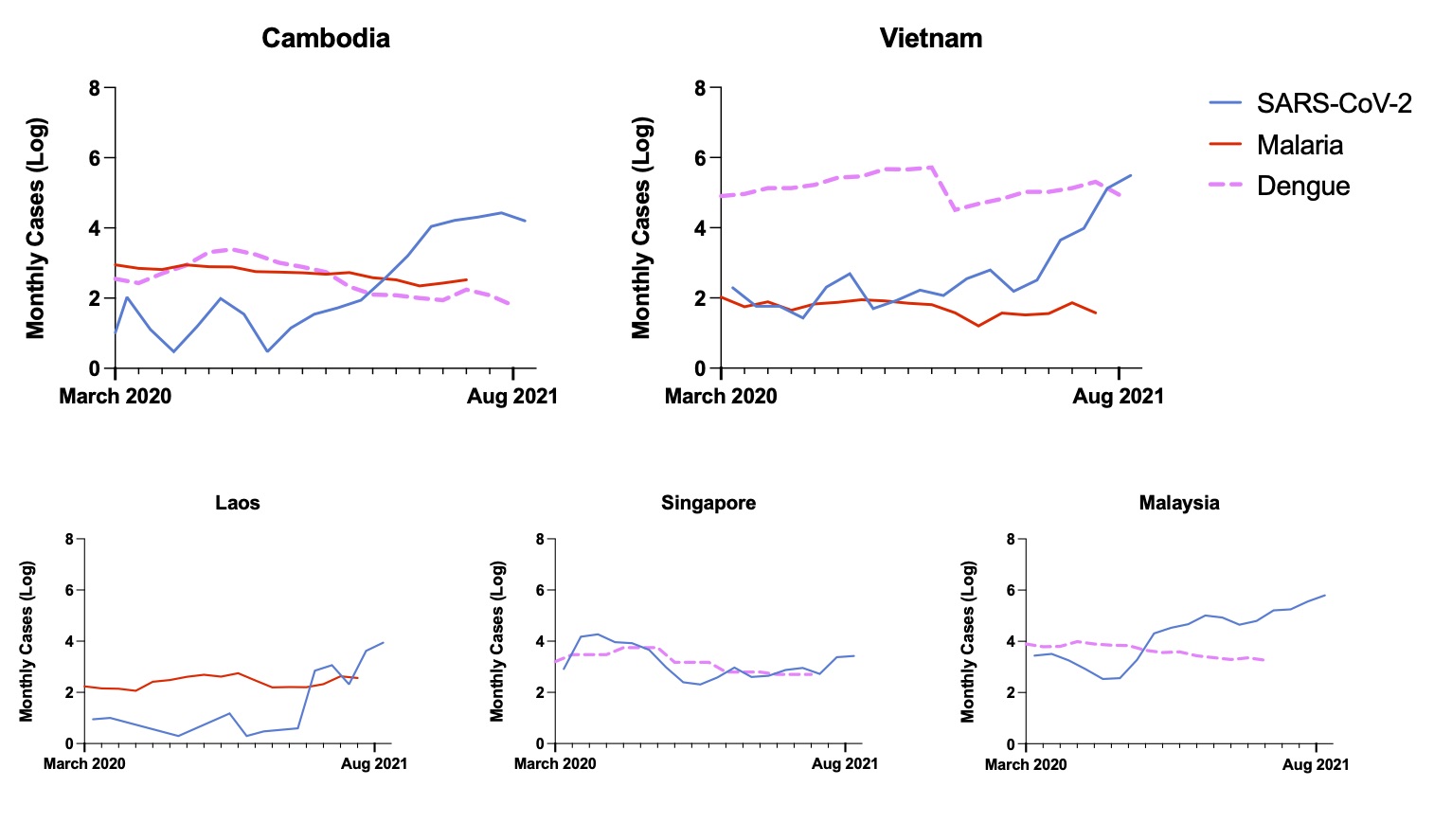

Supplement: Supplementary Figure — | Monthly totals of new cases of SARS-CoV-2, malaria, and dengue from March 2020 through August 2021. Data on SARS-CoV-2 obtained from the Global Change Data Lab (URL: https://covid.ourworldindata.org/, accessed September 9, 2021). Data on malaria and dengue obtained from individual country Ministry of Health public surveillance systems; this was only available for Cambodia and Vietnam (both malaria and dengue data available), Laos (malaria data only), and Malaysia and Singapore (dengue data only). [file NIHMS1790219-supplement-Supplementary_Figure.jpeg]
